# Mediation pathways for reduced substance use among parents in South Africa: a randomized controlled trial

**DOI:** 10.1186/s12889-021-11651-6

**Published:** 2021-09-10

**Authors:** Adeem Ahmad Massarwi, Lucie Cluver, Franziska Meinck, Jenny Doubt, Jamie M. Lachman, Yulia Shenderovich, Ohad Green

**Affiliations:** 1 Centre for Evidence-Based Intervention, Department of Social Policy & Intervention, University of Oxford, Oxford, UK; 2 Department of Social Work, Ben-Gurion University of the Negev, Beersheba, Israel; 3 Department of Psychiatry and Mental Health, University of Cape Town, Cape Town, South Africa; 4 School of Social & Political Sciences, University of Edinburgh, Edinburgh, UK; 5 Faculty of Health Sciences, North-West University, Vanderbijlpark, South Africa; 6 MRC/CSO Social and Public Health Sciences Unit, University of Glasgow, Glasgow, UK; 7 Wolfson Centre for Young People’s Mental Health, Cardiff University, Cardiff, UK; 8 Centre for the Development and Evaluation of Complex Interventions for Public Health Improvement (DECIPHer), School of Social Sciences, Cardiff University, Cardiff, UK

**Keywords:** Substance use, Parenting intervention, Parental depression

## Abstract

**Background:**

Substance use is a major public health concern worldwide. Alcohol and drug use have increased during recent decades in many low- and middle-income countries, with South Africa, where this study was conducted, having among the highest rates in the world. Despite existing evidence on the effectiveness of family-based interventions in reducing substance use among parents and caregivers in low- and middle-income countries, little is known about the mechanism of change that contributes to the reduction. This study investigated mediators of change in a parenting programme (Parenting for Lifelong Health [PLH]) on reducing substance use among parents and caregivers of adolescents through three potential mediators: parental depression, parenting stress and family poverty.

**Methods:**

The current study used a pragmatic cluster randomized controlled trial design. The total sample comprised 552 parent and caregiver of adolescents M = 49.37(SD = 14.69) who were recruited from 40 communities in South Africa’s Eastern Cape. Participants completed a structured confidential self-report questionnaire at baseline and a follow-up test 5 to 9 months after the intervention. Structural equation modeling was conducted to investigate direct and indirect effects.

**Results:**

Analyses indicated that the effect of the PLH intervention on reducing parental substance use was mediated in one indirect pathway: improvement in parental mental health (reduction in parental depression levels). No mediation pathways from the PLH intervention on parental substance use could be associated with parenting stress or family poverty.

**Conclusions:**

The findings of the study suggest that intervention approaches targeting mental health among parents and caregivers have promise for reducing parental substance use. These findings emphasize the need to create supportive environments and systems for parents who suffer from emotional strain and mental health problems, particularly within families experiencing adversity.

**Trial registration:**

Pan-African Clinical Trials Registry PACTR201507001119966. Registered on 27 April 2015. The trial can be found by searching for the key word ‘Sinovuyo’ on the Pan-African Clinical Trials Registry website or via the following link: http://www.pactr.org/ATMWeb/appmanager/atm/atmregistry?_nfpb=true&_windowLabel=BasicSearchUpdateController_1&BasicSearchUpdateController_1_actionOverride=%2Fpageflows%2Ftrial%2FbasicSearchUpdate%2FviewTrail&BasicSearchUpdateController_1id=1119

## Background

Substance use is a major public health concern worldwide [1, 2]. Whereas there is significant variation in levels of substance use globally, alcohol and drug use has increased during recent decades in many low-income countries [1–3]. For example, a national survey conducted among a representative sample of South African adults has shown an increase in drug use over the past decade [4]. Furthermore, and despite the fact that developed countries report the highest alcohol consumption levels globally [5], problematic alcohol use in South Africa ranks among the highest not only in Africa, but in the world [6–8]. Furthermore, a study conducted among 1115 adult men in Cape Town, South Africa, showed that most (75%) reported having engaged in heavy alcohol intake at least once during the preceding week [9].

Empirical studies have shown that substance use among adults is associated with physical, mental, and social problems [7, 10]. For example, substance use is strongly associated with interpersonal violence and injuries [11]. Also, it is one of the main contributors to morbidity and mortality in South Africa [12]. In addition, it has been found that alcohol abuse is a significant risk factor for infectious diseases, such as HIV [6], as well as for involvement in high-risk sexual behaviors [13, 14].

Although previous studies show high rates of substance use among South African adults, the availability of treatment services and interventions that target substance use are still limited, which places South African families at greater risk for suffering from the harmful consequences of substance use [7]. Therefore, there is a compelling need to investigate the effectiveness of interventions that target substance use among parents in South Africa, particularly when the effects of substance use extend beyond the individual users and affect the well-being of the entire family.

Existing evidence from low- and middle-income countries has suggested the effectiveness of family-based interventions on the reduction of substance use among parents and caregivers. For example, a mixed-method randomized controlled study conducted among 61 HIV-affected caregivers in post-genocide Rwanda found that a family-based intervention (the Family Strengthening Intervention for HIV-affected Families) that addresses intimate family violence among HIV-affected families was found effective in reducing alcohol use among caregivers [15]. Similarly, findings of a randomized controlled trial of a parenting programme that combined parenting and economic strengthening components and addressed child maltreatment in South Africa (Parenting for Lifelong Health for Parents and Teens programme – Sinovuyo Teen) found the programme to be effective at reducing substance use among parents/caregivers. The trial was conducted among 552 families with adolescents (aged 10–18 years) in the Eastern Cape, South Africa. At 5 to 9 months after the intervention, the intervention was associated with lower levels of substance use among parents/caregivers [16].

Despite this indication of the effectiveness of family-based interventions in reducing substance use among parents and caregivers in low and middle-income countries, little is known about the mechanism of change that may contribute to substance use reduction. To the best of our knowledge, no research has yet examined the mechanisms of substance reduction among parents and caregivers for family-based interventions. Therefore, the aim of the current study was to investigate the mechanism of substance use reduction among parents and caregivers through three potential mediators: parenting stress, parental depression and family poverty.

Parenting stress has been identified as the pressure parents experience as a result of everyday challenges associated with childrearing, especially when the parents have inadequate resources for meeting their responsibilities as caregivers [17, 18]. The findings of previous studies indicate that parenting stress increases adults’ susceptibility to using substances as a coping mechanism with stressful interactions with their children [19]. Similarly, another study has found that mothers with a substance use disorder can benefit from interventions aimed at reducing parenting stress [20]. Therefore, we assumed that improvements in parenting stress would contribute to a reduction in substance use among parents.

Previous studies investigating the association between parental depression and substance use have found a significant positive association between depressed moods and substance and alcohol use among adults [21–23]. One possible explanation for the relationship between depression and substance use is that substance use is a mechanism of coping with dysphoric moods [24]. For example, a study conducted among 1910 African American adults showed that turning to substance use is a means of alleviating depression stemming from stressful life events [25]. Based on these findings, we assumed that improvements in parental mental health (lower levels of depression) would contribute to a reduction in parental substance use.

In addition, previous studies have shown that lower socioeconomic status has been linked to increased substance use among adults [26]. For instance, a study conducted among 1357 young adult people in South Africa indicated that economic hardship and food insecurity are likely to be related to high levels of alcohol and drug use [27]. Disadvantaged Kenyan fathers who participated in a qualitative study reported that supporting their families financially was a motivator to attempt to cease their alcohol use and problem drinking [28]. Therefore, we assumed that improving household economic status would contribute to a reduction in substance use among parents.

**Fig. 1:**
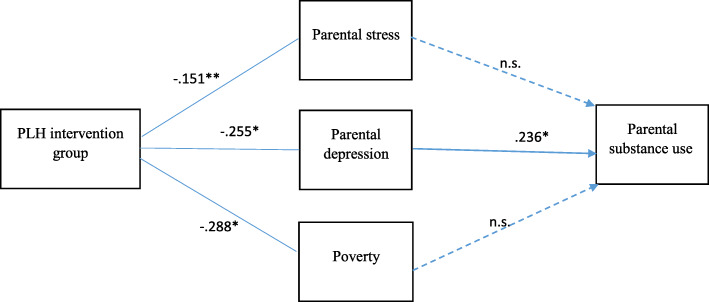
Study model and structural equation model results. Note: All the paths were predicted; those represented by a dotted line were not statistically significant (χ^2^ = 396.28; df = 159; *P* < .000; comparative fit index = .904; root mean standard error of approximation = .049). The mediators and outcome were measured at the same time point (the follow-up test at 5 to 9 months). *P < .00., **P < .050

The current study investigated the mechanism of a parenting programme (PLH) on the reduction of substance use among parents and caregivers of adolescents through three potential mediators: parenting stress, parental depression, and family poverty. Based on the model shown in Fig. 1, we hypothesized that (1) PLH intervention would reduce parenting stress, parental depression, and family poverty; and (2) reduction in parenting stress, parental depression, and family poverty would mediate the association between the PLH intervention and reduction of substance use among parents.

## Methods

### Study design and sample

In this pragmatic cluster randomized controlled trial, the total sample comprised 552 parents and caregivers of adolescents M = 49.37 (SD = 14.69) who were recruited from 40 communities (located in 34 rural villages and three large peri-urban townships) in South Africa’s Eastern Cape. Further information about the study design and sample and the inclusion and exclusion criteria is available in Cluver et al. [29].

Randomization was stratified by urban location and conducted after the baseline pretest using a random number generator by an independent, blinded statistician (CL). Complete randomization within strata used a 1:1 ratio of intervention: control. The sample included 270 families in the intervention arm and 282 families in the control arm, with a mean (SD) of 14 (1.9) families per cluster. Blinding of participants and research staff was not feasible for parenting programmes.

Ethical approval was granted by the University of Oxford (SSD/CUREC2/11–40), the University of Cape Town (PSY2014–001), and the South African Eastern Cape Provincial Departments of Social Development and Education.

### Procedure and data collection

Parents and caregivers completed a structured self-report questionnaire at two points in time: pretest (baseline) and 5 to 9 months after the intervention. Programme implementation and post-test data collection were delayed as the result of extended political and civil violence. The final data collection stage was originally intended to be at 12 months post-intervention, but because of political violence and funding constraints was only able to begin at 5 months post-intervention and took 5 months to be completed due to the study sample size and spread across both rural and urban sites. That was the reason for the wide range of the post-intervention assessments.

### Intervention group

Parents and caregivers in the intervention group participated in a 14-session parenting programme, ‘Parenting for Lifelong Health/Sinovuyo Teen’. Each session lasted for 1 to 1.5 h a week. All sessions took place in public and community locations, such as churches, community halls and schools, or outdoors, under trees.

Based on Social Learning Theory [30], the programme included a set of 14 psychosocial sessions designed to improve the parent-child relationship and family cohesion and harmony, to promote non-violent discipline, and to encourage the family members to spend quality time together. In addition to the parent-child relationship, the programme also emphasized certain parenting principles as important for maintaining healthy family relationships, such as complimenting each other, engaging in joint problem-solving, implementing rules and routines, responding to crises together, establishing clear communication strategies, and applying mindfulness practices to reduce stress and anger levels. For example, mindfulness practices included taking a pause – a brief breath-awareness activity – and a body relaxation exercise in which participants gave focused attention to each part of their body, aimed at reducing stress. Participants practiced mindfulness movement exercises at the beginning of each session.

All sessions used collaborative problem-solving techniques (not didactic methods), traditional stories, role-play, modelling, and stress-reduction activities. In addition to its psychosocial elements, the programme also included core economic components designed to improve families’ financial status, including: (1) encouraging families to save some of their earnings through presenting a short play that addressed common financial challenges; (2) teaching fundamental financial skills, such as budgeting and saving through visual budgeting exercises; and (3) motivating mental commitment to saving by clearly defining family saving goals and making a practical family financial plan. The programme was designed for low-resource settings with no technology (such as video) or literacy requirements. For further details about the programme, please see Cluver et al. [29].

Participants were encouraged to engage in home practice in the week following each session. For participants unable to attend sessions because of illness or disability, make-up meetings were arranged at home or in the hospital to provide brief session content. A simple lunch was included at the beginning of each session because many participants found it difficult to concentrate owing to hunger. The programme was delivered by local community members who were trained by a local non-governmental organization (NGO), Clowns Without Borders South Africa, and supported through weekly supervision.

### Control group

Parents/caregivers in the control group received one five-hour session of a hygiene programme called ‘SinoSoap’, conducted by the Clowns without Borders NGO in South Africa. The control condition involved drama-based skill building, delivered through performances and activities, about conserving safe water and children’s handwashing. Thus, the control condition was not related to parenting practice; instead, it addressed hygiene and sanitation handwashing activities to increase the likelihood of retention in the control group. This control activity was unlikely to influence any primary or secondary outcomes.

### Measurements

Parents/caregivers completed self-report questionnaires using tablets at baseline and at 5 to 9 months after the intervention. All questionnaires were pre-piloted with local parents/caregivers of adolescents. All measurements were translated into isiXhosa, one of the 11 official languages spoken in South Africa, and then back-translated.

Alcohol and substance use among parents/caregivers were assessed by using the adapted version of the World Health Organization (WHO) Alcohol Use Disorders Identification Test (AUDIT) [31] and the WHO Global School-based Health Survey. This variable was reported by parents/caregivers and used three items (α = .86), such as ‘In the past month, have you had a drink?’ and ‘Did you take any drugs to help you relax?’ Responses were scored as 0 = *no* and 1 = *yes*. One overall score was derived by computing the sum of the items.

Parenting stress was measured using 18 items (α = .77) from the Parental Stress Scale [32], such as ‘I feel overwhelmed by the responsibility of being a parent’ and ‘Caring for my children sometimes takes more time and energy than I have to give’. Eight items from the scale were reverse coded (‘I am happy in my roles as a parent’, ‘I am satisfied as a parent’, ‘I find my child (ren) enjoyable’, ‘I enjoy spending time with my child (ren)’, ‘My child (ren) is (are) an important source of affection for me’, ‘Having children gives me a more certain and optimistic view of the future’, ‘I feel close to my child (ren), and ‘There is little or nothing I wouldn’t do for my child (ren) if it was necessary’). Items were measured on a 5-point Likert-type scale, ranging from 0 (*strongly disagree*) to 4 (*strongly agree*). An overall score was derived by computing the sum of the items.

Parental depression was assessed by using 20 items (α = .87) from the Centre for Epidemiological Studies Depression Scale [33], such as ‘I felt very sad even with help from my family and friends’, ‘I didn’t feel like eating’, and ‘My appetite was poor’. Four items from the scale were reverse coded (‘I enjoyed life’, ‘I was happy’, ‘I felt hopeful about the future’, and ‘I felt I was just as good as other people’). Items were measured on a 5-point Likert-type scale, ranging from 0 (*not at all* or *less than once a day*) to 4 (*nearly every day*). An overall score was derived by computing the sum of the items.

Family poverty was measured as monthly consistent access to necessities, including food, electricity, communication, and transport [34]. This variable was assessed by using 8 items (α = .71) such as ‘Afford three meals a day’, ‘Afford the costs of the school’ and ‘Afford enough warm clothes’. Responses were 0 = *no* and 1 = *yes*. One overall score was derived by computing the sum of the items.

### Covariates

Parents\caregivers were asked to provide information about their age, gender, and rural or urban location.

### Data analyses

Analyses used intention-to-treat for all clusters and families irrespective of intervention uptake and included families who were no longer living together at follow-up (*n* = 53). Independent-sample *t* tests were conducted to compare the means of outcomes and mediator differences between the intervention and control groups at baseline and follow-up. All variables (mediators and outcomes) were measured at baseline and at the follow-up 5 to 9 months after the intervention was completed.

A linear structural equation model (SEM) was used with the AMOS 21 statistics program. The SEM procedure combined measurement modeling (confirmatory factor analyses) and SEM. Items that were theoretically and empirically perceived as describing the variable were used in the measurement model.

Goodness of fit for the final model was assessed using the comparative fit index (CFI; acceptable fit for CFI is ≥ .90) and the root mean standard error of approximation (RMSEA – acceptable fit for RMSEA is < .06). We also report χ^2^ fit statistics but acknowledge that the test result is inflated by the sample size of the study.

## Results

### Descriptive statistics

The *t* test results for baseline and follow-up outcomes and mediating variables (intervention and control group) are shown in Table 1.

**Table 1:** Baseline and follow-up characteristics for intervention and control groups

	Baseline Mean (SD)		Follow-Up Mean (SD)	
Variable	Treatment	Control	Treatment	Control
**Parental substance use**	0.44 (.85)	0.56 (.93)	0.34* (0.75)	0.60 (1.02)
**Parental depression**	23.13 (11.79)	24.90 (12.08)	11.30* (9.78)	16.82 (11.13)
**Parenting stress**	33.13 (8.68)	33.39 (8.18)	23.75* (8.24)	27.05 (7.32)
**Family poverty**	0.04 (1.68)	−.004 (1.64)	0.29 (1.60)*	−0.28 (1.49)
**N**	270	282	264	278

*Statistically significant differences in means between the treatment and control groups at *P* < .05

### Direct and indirect effects

We examined three potential mediators (parenting stress, parental depression, and family poverty) of the effect of PLH intervention on reduction of substance use among parents/caregivers at the follow-up test (5 to 9 months after the intervention).

Table 2 shows the total, direct and indirect effect of each mediator on the outcome of the study. At the first step of the analyses, each mediator was tested individually. At the second step, all mediators were tested in an SEM simultaneously.

**Table 2:** Total, direct and indirect effects of mediators on substance use among parents

	Parental substance use		
Mediators	Total Effect	Direct Effect	Indirect Effect
1. Parental depression	−.246 [−.39,-.10]	−.201 [−.35,-.05]	−.044 [−.11,-.01]
2. Parenting stress	−.250 [−.40,-.09]	−.217 [−.37,-.06]	−.033 [−.07,-.00]
3. Family poverty	−.205 [−.33,-.07]	−.208 [−.39,-.09]	.001 [−.03,.03]

In the results of the measurement-fit model, the values of the CFI (.931) and RMSEA (.043) showed a good model fit (χ^2^ = 284.89; df = 142; *P* < .000). Structural equation modeling was also used to test the direct and indirect (mediation) effects of the PLH intervention and the potential mediators on substance use among parents/caregivers. The model shown in Fig. 1 represents the model fit for all the variables of the study. In the results of the theoretical model, the values of the CFI (.904) and RMSEA (.049) showed a good model fit (χ^2^ = 369.28; df = 159; *P* < .000).

The PLH intervention had a significant effect on reducing parental depression (β = −.255; *P* < .001), parenting stress (β = −.151; *P* < .05) and family poverty (β = −.288; *P* < .001) at the follow-up test (5 to 9 months after the intervention).

Mediation was examined using Bootstrap in AMOS. The results presented in Fig. 1 indicate that the PLH intervention’s effect on reducing parental substance use among parents/caregivers was associated with one indirect mediation pathway: reduction in parental depression at the follow-up test (5 to 9 months after the intervention) (β = −.255; 95%, CI = − 11 to .01; P < .001). There were no mediation pathways from the PLH intervention to parental substance use through parenting stress or family poverty.

## Discussion

The current study investigated the role of parental depression, family poverty and parenting stress as potential mediators of the PLH parenting programme in reduction of substance use among parents and caregivers of adolescents in South Africa. The findings of the study contribute to understanding the mechanism behind the reduction of substance use among parents by showing that a reduction in parental depression served as a mediator between the PLH intervention effect and parental substance use. Consequently, improving parental mental health by reducing depression was associated with a reduction in substance use among parents and caregivers.

This mediation process can be interpreted in light of Agnew’s General Strain Theory [35], according to which, substance use among adults is a coping mechanism to relieve negative feelings such as stress, frustration and depression. With limited support and skills, parents may resort to substance use to escape their pain and negative feelings and to cope with the problems they face. The study’s findings suggest that PLH intervention provides parents with skills and support that help them to cope in effective ways and to avoid ineffective coping mechanisms such as problematic alcohol and drug use. The PLH intervention contributed positively to parents’\caregiver’ mental health by providing emotional and instrumental support as part of the intervention (such as stress-reduction activities that included deep-breath awareness activities and body relaxation exercises in which participants gave attention to each part of their body). This finding is consistent with previous studies showing that mindfulness practices, which can reduce depressive symptoms, are effective approaches in reducing substance use [36]. One explanation for the potential effectiveness of mindfulness practices in reducing substance use is that these practices may increase awareness of physical, emotional, and cognitive states, which may contribute to a decrease the need to alleviate feelings of discomfort with substance use and may encourage more mindful ways to cope with emotional difficulties [37].

Contrary to our hypotheses, the findings of this study showed that improvement in family poverty and parenting stress did not mediate the PLH intervention effect on reduction in substance use among parents. Despite evidence that family poverty is a risk factor for substance use [26, 27], the findings of this study showed that improvement in the financial status of the family did not mediate the association between the PLH intervention and reduction of substance use among parents at the follow-up intervention. In other words, improvement in the economic status of the family did not explain the reduction in parental substance use at the follow-up intervention. This result could be attributable to the fact that mediation analyses were conducted at one time point only. As changes in family economic status are affected by financial behaviors of parents and psychosocial factors [38], we suggest that improvement in economic status over a sustained time could have a significant effect on reduction of parental substance use, particularly in the context of chronic economic hardship.

With respect to parenting stress, contrary to our hypothesis, reduction in parental stress levels did not mediate the association between the impact of the PLH intervention and reduction in substance use among parents at the follow-up intervention. One explanation is that previous studies focused mainly on tobacco-smoking parents who described smoking as a source of relief during stressful interactions with their children [39]. The current study did not examine tobacco smoking among parents and focused on other substances, such as drugs and heavy alcohol drinking. Therefore, we could expect different mechanisms for different substances which could possibly explain the nonsignificant effect of improving parenting stress on reducing parental substance use among the current study’s sample. In addition, previous studies focused mainly on the impact of parenting stress on substance use reduction among mothers of young children [40, 41], which does not necessarily reflect the experience of parents and caregivers of adolescents, due to different needs and demands when parenting older children. Therefore, future research is needed to examine different mechanisms by which engagement in parenting interventions contributes to reductions in substance use among parents of adolescents.

To the best of our knowledge, the current study is among the first to investigate mediation pathways for reduction in substance use among parents and caregivers in low- to middle-income countries. Strengths of the study include the pragmatic randomized trial method, which provides high external validity. Furthermore, standardized measurement and intention-to-treat were used. However, limitations also need to be acknowledged. First, mediation analyses were conducted at one time point only (after 5 to 9 months of follow-up). A longer-term follow-up with multiple post-intervention assessments would have enabled us to examine potential effects and potential reverse causality between parental depression and reduction of parental substance use. Hence, future studies should conduct mediation analyses at more than one point in time, which would enable the hypothesized mediator to be measured before the outcome. Second, based on the findings of the study, causal inferences of intervention components cannot be drawn. The findings of the study suggest that improvement in parental mental health (less depression) mediates parental substance use reduction. However, we cannot recognize which intervention components are responsible for this mediation effect. Therefore, it is recommended that future studies use other methods of identifying essential components, such as relaxation and skills for coping with negative feelings, which might provide further insight into active core ingredients for parenting programs. This includes evidence from randomized micro-trials on the efficacy of discrete parenting techniques [42] and factorial experiment trials that test different components in relation to each other [43].

## Conclusions

The findings of the current study emphasize the importance of understanding the challenges vulnerable parents and caregivers face that negatively affect their mental health and may increase the likelihood of involvement in high-risk behaviors such as substance use. These findings highlight the need to create supportive environments and systems for parents who suffer from emotional strain and mental health problems. Professionals need to adopt an empathic approach toward vulnerable parents, which would contribute to better understanding their needs and challenges and to building effective psychosocial interventions and prevention programs that target families at risk.

## Data Availability

Sinovuyo Teen manuals and programme materials will be made freely available online, and UNICEF has sponsored free printed versions. All research materials (i.e., questionnaires, study process materials and a qualitative toolkit) will be made freely available on the UNICEF and WHO websites. The study data will be made available on open-access websites such as the South African Data Archive and the European Clinical Trials database. Further information about the protocol study is available at 10.1186/s13063-016-1452-8.

## Abbreviations

AUDIT: Alcohol Use Disorders Identification Test
CFI: Comparative Fit Index
NGO: non-governmental organisation
PLH: Parenting for Lifelong Health
RMSEA: root mean standard error of approximation
SEM: structural equation model
WHO: World Health Organization

## References

[1] Degenhardt L, Whiteford HA, Ferrari AJ, Baxter AJ, Charlson FJ, Hall WD, Freedman G, Burstein R, Johns N, Engell RE, Flaxman A, Murray CJL, Vos T (2013). Global burden of disease attributable to illicit drug use and dependence: findings from the global burden of disease study. Lancet.

[2] McGovern R, Addison MT, Newham JJ, Hickman M, Kaner EFS (2017). Effectiveness of psychosocial interventions for reducing parental substance misuse. Cochrane Database Syst Rev.

[3] Scheibe A, Shelly S, Versfeld A, Howell S, Marks M. Safe treatment and treatment of safety: call for a harm-reduction approach to drug-use disorders in South Africa. South African Health Review. 2017 1;2017(1):197–204.

[4] Peltzer K, Phaswana-Mafuya N (2018). Drug use among youth and adults in a population-based survey in South Africa. S Afr J Psychiatry.

[5] World Health Organization. Global status report on alcohol and health 2014. Geneva, Switzerland: World Health Organization; 2014. Available from: http://apps.who.int/iris/bitstream/10665/112736/1/9789240692763.eng.pdf?ua=1 [Accessed January 2015].

[6] World Health Organization. Global status report on alcohol and health 2018. Geneva: World Health Organization; 2018. Available from: https://www.who.int/publications/i/item/9789241565639 [Accessed 24 June 2021].

[7] Groenewald C, Bhana A (2018). Substance abuse and the family: an examination of the south African policy context. Drugs-Educ Prev Policy.

[8] Rehm J, Mathers C, Popova S, Thavorncharoensap M, Teerawattananon Y, Patra J (2009). Global burden of disease and injury and economic cost attributable to alcohol use and alcohol-use disorders. Lancet..

[9] Arfer KB, Tomlinson M, Mayekiso A, Bantjes J, van Heerden A, Rotheram-Borus MJ (2018). Criterion validity of self-reports of alcohol, cannabis, and methamphetamine use among young men in Cape Town, South Africa. Int J Ment Health Addiction.

[10] Myers B, van der Westhuizen C, Naledi T, Stein DJ, Sorsdahl K (2016). Readiness to change is a predictor of reduced substance use involvement: findings from a randomized controlled trial of patients attending south African emergency departments. BMC Psychiatry.

[11] Seedat M, Van Niekerk A, Jewkes R, Suffla S, Ratele K (2012). Violence and injuries in South Africa: prioritising an agenda for prevention. Lancet..

[12] Mayosi BM, Lawn JE, van Niekerk A, Bradshaw D, Abdool Karim SS, Coovadia HM (2012). Health in South Africa: changes and challenges since 2009. Lancet..

[13] Chawla N, Sarkar S (2019). Defining “high-risk sexual behavior” in the context of substance use. J Psychosexual Health.

[14] Bello B, Moultrie H, Somji A, Chersich MF, Watts C, Delany-Moretlwe S (2017). Alcohol use and sexual risk behaviour among men and women in inner-city Johannesburg. South Africa BMC Public Health.

[15] Chaudhury S, Brown FL, Kirk CM, Mukunzi S, Nyirandagijimana B, Mukandanga J, Ukundineza C, Godfrey K, Ng LC, Brennan RT, Betancourt TS (2016). Exploring the potential of a family-based prevention intervention to reduce alcohol use and violence within HIV-affected families in Rwanda. AIDS Care.

[16] Cluver L, Meinck F, Steinert J, Shenderovich Y, Doubt J, Romero R (2018). Parenting for lifelong health: a pragmatic cluster randomised controlled trial of a non-commercialised parenting programme for adolescents and their families in South Africa. BMJ Glob Health.

[17] Cousino MK, Hazen RA (2013). Parenting stress among caregivers of children with chronic illness: a systematic review. J Pediatr Psychol.

[18] Pereira J, Vickers K, Atkinson L, Gonzalez A, Wekerle C, Levitan R (2012). Parenting stress mediates between maternal maltreatment history and maternal sensitivity in a community sample. Child Abuse Negl.

[19] Rutherford HJ, Mayes LC (2019). Parenting stress: a novel mechanism of addiction vulnerability. Neurobiol Stress.

[20] Short VL, Gannon M, Weingarten W, Kaltenbach K, LaNoue M, Abatemarco DJ (2017). Reducing stress among mothers in drug treatment: a description of a mindfulness based parenting intervention. Matern Child Health J.

[21] Conner KR, Pinquart M, Gamble SA (2009). Meta-analysis of depression and substance use among individuals with alcohol use disorders. J Subst Abus Treat.

[22] Davis EC, Rotheram-Borus MJ, Weichle TW, Rezai R, Tomlinson M (2017). Patterns of alcohol abuse, depression, and intimate partner violence among township mothers in South Africa over 5 years. AIDS Behav.

[23] Mossie A, Kindu D, Negash A (2016). Prevalence and severity of depression and its association with substance use in Jimma town. Southwest Ethiopia Depress Res Treat.

[24] Rappeneau V, Bérod A (2017). Reconsidering depression as a risk factor for substance use disorder: insights from rodent models. Neurosci Biobehav Rev.

[25] Clark TT (2014). Perceived discrimination, depressive symptoms, and substance use in young adulthood. Addict Behav.

[26] Lee JO, Cho J, Yoon Y, Bello MS, Khoddam R, Leventhal AM (2018). Developmental pathways from parental socioeconomic status to adolescent substance use: alternative and complementary reinforcement. J Youth Adolesc.

[27] Gibbs A, Jewkes R, Willan S, Washington L (2018). Associations between poverty, mental health and substance use, gender power, and intimate partner violence amongst young (18-30) women and men in urban informal settlements in South Africa: a cross-sectional study and structural equation model. PLoS One.

[28] Patel P, Kaiser BN, Meade CS, Giusto A, Ayuku D, Puffer E (2020). Problematic alcohol use among fathers in Kenya: poverty, people, and practices as barriers and facilitators to help acceptance. Int J Drug Policy.

[29] Cluver L, Lachman JM, Ward C, Gardner F, Petersen T, Meinck F, et al. Development of a parenting support programme to prevent abuse of adolescents in South Africa: findings from a pilot pre-post study. Research Soc Work Pract 2016;doi:10.1177/1049731516628647, 2017.

[30] Bandura A, Walters RH (1977). Social learning theory.

[31] Saunders JB, Aasland OG, Babor TF, De la Fuente JR, Grant M (1993). Development of the alcohol use disorders identification test (AUDIT): WHO collaborative project on early detection of persons with harmful alcohol consumption-II. Addiction.

[32] Berry JO, Jones WH (1995). The parental stress scale: initial psychometric evidence. J Soc Pers Relat.

[33] Radloff LS (1977). The CES-D scale: a self-report depression scale for research in the general population. Appl Psychol Meas.

[34] Morduch J (1995). Income smoothing and consumption smoothing. J Econ Perspect.

[35] Agnew R (2001). Building on the foundation of general strain theory: specifying the types of strain most likely to lead to crime and delinquency. J Res Crime Delinq.

[36] Chiesa A, Serretti A (2014). Are mindfulness-based interventions effective for substance use disorders? A systematic review of the evidence. Subst Use Misuse.

[37] Bowen S, Chawla N, Collins SE, Witkiewitz K, Hsu S, Grow J, Clifasefi S, Garner M, Douglass A, Larimer ME, Marlatt A (2009). Mindfulness-based relapse prevention for substance use disorders: a pilot efficacy trial. Subst Abuse.

[38] Steinert JI, Cluver LD, Meinck F, Nzima D, Doubt J (2020). Opening the black box: a mixed-methods investigation of social and psychological mechanisms underlying changes in financial behaviour. J Dev Stud.

[39] Correa JB, Simmons VN, Sutton SK, Meltzer LR, Brandon TH (2015). A content analysis of attributions for resuming smoking or maintaining abstinence in the post-partum period. Matern Child Health J.

[40] Moreland AD, McRae-Clark A (2018). Parenting outcomes of parenting interventions in integrated substance-use treatment programs: a systematic review. J Subst Abus Treat.

[41] Suchman N, Pajulo M, DeCoste C, Mayes L (2006). Parenting interventions for drug-dependent mothers and their young children: the case for an attachment-based approach. Fam Relat.

[42] Leijten P, Dishion TJ, Thomaes S, Raaikmakers MAJ, Orobio de Castro B, Mattys W (2015). Bringing parenting interventions back to the future: how randomized controlled microtrials may benefit parenting intervention effectiveness. Clin Psychol Sci Pract.

[43] Collins LM, Murphy SA, Nair VN, Strecher VJ (2005). A strategy for optimizing and evaluating behavioral interventions. Ann Behav Med.

